# Plasma Vitamin D and Biomarkers of Cardiometabolic Disease Risk in Adult Canadians, 2007–2009

**DOI:** 10.5888/pcd10.120230

**Published:** 2013-06-06

**Authors:** Bibiana García-Bailo, Laura A. Da Costa, Paul Arora, Mohamed Karmali, Ahmed El-Sohemy, Alaa Badawi

**Affiliations:** Author Affiliations: Bibiana García-Bailo, Mohamed Karmali, Department of Nutritional Sciences, University of Toronto, and Office of Biotechnology and Population Health, Public Health Agency of Canada, Toronto, Canada; Laura A. Da Costa, Ahmed El-Sohemy, Department of Nutritional Sciences, University of Toronto, Toronto, Canada; Paul Arora, Office of Biotechnology and Population Health, Public Health Agency of Canada, and Dalla Lana School of Public Health, University of Toronto, Toronto, Canada.

## Abstract

**Introduction:**

Vitamin D may modulate cardiometabolic disease risk, although the relationship has not been investigated in the general Canadian population. Understanding this relationship may inform public health strategies to curb the incidence of cardiometabolic disease in Canada and elsewhere. The objectives of this study were to examine the association between vitamin D and traditional and novel biomarkers of cardiometabolic disease and to describe the extent of the month-to-month fluctuations of vitamin D in the Canadian population.

**Methods:**

We examined the association between plasma 25-hydroxyvitamin D and a range of cardiometabolic risk biomarkers in participants (n = 1,928; age range, 16–79 years) from the Canadian Health Measures Survey. We conducted linear regressions analyses (adjusted for sex, waist circumference, physical activity, hormone use, and season) to assess the relationship between 25-hydroxyvitamin D and biomarkers of dysglycemia, dyslipidemia, and inflammation in the study population. We repeated analyses stratified by sex, and we evaluated monthly fluctuations in 25-hydroxyvitamin D in men and women.

**Results:**

We observed wide month-to-month variations in 25-hydroxyvitamin D; fluctuations were more pronounced in men. Plasma 25-hydroxyvitamin D was inversely associated with insulin, insulin resistance, triglycerides, total cholesterol, low-density lipoprotein cholesterol, and the ratio of total to high-density lipoprotein cholesterol but not with fasting glucose, apolipoprotein A1, apolipoprotein B, C-reactive protein, fibrinogen, or homocysteine. This pattern varied between men and women.

**Conclusion:**

Vitamin D may modulate various metabolic processes and may influence cardiometabolic disease risk in Canadians. These findings may have public health implications when recommending vitamin D for the prevention of cardiometabolic disease and related conditions.

## Introduction

Cardiometabolic disease is characterized by dyslipidemia, dysglycemia, abdominal obesity, and hypertension (1). Novel biomarkers of risk, such as apolipoprotein (Apo) A1 and ApoB (2), and the inflammatory markers C-reactive protein (CRP), fibrinogen, and homocysteine (3–5), have been proposed. Inadequate vitamin D status has been associated with elevated cardiometabolic disease risk, although results are inconsistent, as evidenced by recent meta-analyses pooling multiple study populations (6–8). These inconsistencies may result partly from differences in sample size, dosage, geographic location, and disease progression across studies.

The prevalence of vitamin D insufficiency (plasma 25-hydroxyvitamin D [25(OH)D] <50 nmol/L) among Canadians is high (9). This high prevalence of insufficiency may result from a decreased ability to produce vitamin D endogenously during the winter months (10). Indeed, the proportion of vitamin D-insufficient individuals in the general Canadian population was higher in winter than in summer (9). Among elderly in the United States, seasonal variations in vitamin D were greater in men than women (11). Whether these sex-based differences have biologic relevance that may translate into effects on health outcomes is unknown. The possibility that the effects of vitamin D on cardiometabolic disease differ by sex warrants further study. Furthermore, describing how vitamin D levels fluctuate month-to-month may provide a basis for implementing public health strategies to increase 25(OH)D levels when deficiency is most acute.

Recent studies suggest an association between vitamin D status, metabolic syndrome, insulin resistance, and obesity among Canadians (12,13). However, the relationship between 25(OH)D and individual biomarkers of cardiometabolic disease has not been explored in the Canadian population, and whether it differs by sex is unknown. Understanding the association between vitamin D and cardiometabolic risk in Canadians may provide a clearer understanding of how this micronutrient modulates cardiometabolic disease and may inform public health strategies to curb the incidence of cardiometabolic disease in Canada and elsewhere.

Our objectives were to examine the association between 25(OH)D and traditional and novel biomarkers of cardiometabolic disease and to describe the extent of the month-to-month fluctuations of 25(OH)D in a representative sample of the general Canadian population.

## Methods

### Study design and population

We used data from the Canadian Health Measures Survey (CHMS) cycle 3.1, a representative sample of Canadians aged 6 to 79 years in whom direct indicators of health and wellness were measured. Details of the sampling design and strategy, methods, and data collection have been published elsewhere (14). Data were collected between March 2007 and February 2009 at 15 sites selected by using a multistage sampling strategy. The sites were chosen to cover 96.3% of the Canadian population, excluding people living on Aboriginal Reserves, on Crown Lands, in institutions, in remote regions, and full-time members of the Canadian Forces. A total of 6,106 households of the 8,772 selected agreed to participate in this study, for a household response rate of 69.6%. From the 7,483 people selected from these households, 6,604 (88.3%) people agreed to respond to the study questionnaire; 5,604 (84.9%) of the 6,604 agreed to provide physical measurements. The national response rate for this survey was 51.7%. All participants provided written informed consent and the study was approved by the Health Canada Research Ethics Board. For this study, we excluded people younger than 16 years and nonfasting responders. This resulted in a sample size of 1,928 people, representing 25,057,060 Canadians.

### Biologic and anthropometric measures

Details of the collection and handling of biological samples have been described elsewhere (15). Samples were analyzed for cardiometabolic biomarkers, including glucose, fasting insulin, CRP, fibrinogen, homocysteine, triglycerides, total cholesterol, high-density lipoprotein cholesterol (HDL-C), low-density lipoprotein cholesterol (LDL-C), and ApoA1 and ApoB at the Health Canada Laboratory, Bureau of Nutritional Sciences, Nutrition Research Division, by using standard operating procedures. We calculated the ratio of total to HDL-C from measured values for total cholesterol and HDL-C. We calculated the homeostasis model of insulin resistance (HOMA-IR) from fasting measures of insulin (µU/mL) and glucose (mmol/mL) as insulin multiplied by glucose and divided by 22.5. Plasma 25(OH)D was measured by chemiluminescence assay by using the Liaison 25-hydroxyvitamin D Total assay (Diasorin, Ltd, Stillwater, Minnesota). Within-run and between-run coefficients of variation (CVs) for this assay ranged between 3.2% and 8.5% and between 6.9% and 12.7%, respectively, based on preliminary testing using external quality controls from BioRad (BioRad Laboratories, Ltd, Mississauga, Ontario) and Diasorin. Samples analyzed by Health Canada were within these ranges.

We derived body mass index (BMI) as weight (kg) divided by height (m^2^) for measured height and weight. Waist circumference was measured by using a measuring tape at the midpoint between the last floating rib and the top of the iliac crest in the midaxillary line. Systolic and diastolic blood pressures were measured by using BP-TRU automated oscillometric devices (BP-TRU Medical Devices, Ltd, Coquitlam, British Columbia). Daily energy expenditure was calculated from self-reported leisure time physical activities during the past 3 months. A person’s self-reported race was classified into 1 of 3 groups: white, Asian (Korean, Filipino, Japanese, Chinese, South Asian, Southeast Asian, Arab, and West Asian), and other (African Canadian, Latin American, and people of mixed ancestry). Self-reported medication use was collected and coded by using the American Hospital Formulary Service drug code. Subjects were dichotomized (yes/no) on the basis of use of hormones and synthetic substitutes in the previous month. The types of medications included in this categorization included hormonal contraceptives, androgens, estrogens, insulin, and thyroid hormones.

### Statistical analyses

We performed all statistical analyses by using survey procedures in SAS (version 9.2, SAS Institute Inc, Cary, North Carolina). We set the α error at .05, and we report 2-sided *P *values. We considered a *P *value of ≤.05 significant. To account for the complex survey design, we applied bootstrap weights for variance estimates, sampling weights for point estimates, and 11 degrees of freedom. We examined the distributions of continuous variables by plotting histograms and natural log or square-root transformed skewed variables as needed before analysis to improve linearity of relationships and normality of residuals. We examined subject characteristics, including biomarkers of cardiometabolic disease, by sex. We used *t* tests to examine the association between continuous variables, and χ^2^ tests to examine race and hormone use by sex. We plotted unadjusted weighted mean plasma 25(OH)D levels by month of clinic visit and by sex on a single graph to examine month-to-month variation in circulating vitamin D levels in men and women. We produced unadjusted weighted mean levels of cardiometabolic biomarkers by quartiles of 25(OH)D. We conducted linear regression models examining the linear association between cardiometabolic biomarkers and plasma levels of 25(OH)D adjusted for sex, waist circumference, physical activity, hormone use, and season of clinic visit to produce β coefficients and the associated *P* values. We dropped age from regression models because of multicolinearity with waist circumference. We also dropped race from the models because of the heterogeneous nature and small sample size of the nonwhite group. We repeated analyses stratified by sex.

## Results

Anthropometric measures and mean levels of physical activity were significantly lower among women than men (*P* ≤ .002) (Table 1). Mean levels of glucose, HOMA-IR, homocysteine, triglycerides, LDL-C, total: HDL-C, and ApoB were significantly lower among women than men; levels of CRP, fibrinogen, HDL-C, total cholesterol, and ApoA1 were significantly higher among women than men (*P* ≤ .05).

**Table 1:** Characteristics of the Study Participants, Canadian Health Measures Survey, 2007–2009^a^

Characteristic	All (n = 1,928)	Men (n = 927)	Women (n = 1,001)	*P* Value^b^
Hormone use, %	15.8 (13.0–18.6)	6.3 (4.4–8.3)	24.9 (20.3–29.5)	< .001
Age, y	43.5 (43.1–43.9)	42.8 (42.0–43.5)	44.2 (43.6–44.8)	.02
**Race,^c^ %**				
White	84.3 (74.5–94.1)	83.7 (72.4–95.0)	84.9 (75.8–94.0)	.72^d^
Asian^e^	11.4 (4.7–18.1)	11.9 (3.8–20.0)	10.9 (4.2–17.6)	
Daily energy expenditure, kcal/kg/d^f^	1.8 (1.6–2.0)	2.0 (1.8–2.3)	1.6 (1.4–1.8)	.002
BMI, kg/m^2g^	26.8 (26.2–27.4)	27.1 (26.5–27.6)	26.6 (25.7–27.5)	.09
Waist circumference, cm^g^	90.2 (88.5–91.9)	94.1 (92.5–95.8)	86.4 (83.9–88.9)	< .001
Systolic blood pressure, mm Hg^g^	111.2 (109.6–112.7)	113.4 (112.0–114.9)	108.9 (106.7–111.2)	< .001
Diastolic blood pressure, mm Hg	70.7 (69.6–71.8)	73.1 (71.9–74.2)	68.4 (67.0–69.9)	< .001
Plasma 25(OH)D, nmol/L^g^	67.5 (64.4–70.6)	66.8 (62.9–70.6)	68.2 (65.3–71.1)	.23
Glucose, mmol/L^g^	5.1 (5.0–5.1)	5.2 (5.1–5.3)	5.0 (4.9–5.0)	< .001
Fasting insulin, pmol/L^g^	70.9 (65.4–76.4)	72.4 (66.2–78.6)	69.5 (61.7–77.2)	.18
HOMA-IR^g^	2.4 (2.2–2.6)	2.5 (2.3–2.7)	2.3 (2.0–2.6)	.05
Triglycerides, mmol/L^g^	1.3 (1.2–1.4)	1.4 (1.3–1.5)	1.3 (1.1–1.4)	.03
Total cholesterol, mmol/L	4.9 (4.7–5.0)	4.8 (4.7–4.9)	4.9 (4.8–5.0)	.04
HDL-C, mmol/L^f^	1.3 (1.3–1.4)	1.2 (1.2–1.2)	1.5 (1.4–1.5)	< .001
LDL-C, mmol/L^f^	3.0 (2.9–3.1)	3.1 (3.0–3.2)	2.9 (2.8–3.0)	.008
Total:HDL-C^g^	3.9 (3.8–4.0)	4.2 (4.1–4.3)	3.5 (3.4–3.7)	< .001
ApoA1, g/L^f^	1.4 (1.4–1.5)	1.3 (1.3–1.4)	1.5 (1.5–1.6)	< .001
ApoB, g/L^g^	0.91 (0.88–0.93)	0.92 (0.89–0.95)	0.89 (0.86–0.92)	.05
CRP, mg/L^g^	2.3 (2.0–2.5)	2.1 (1.8–2.4)	2.5 (2.2–2.7)	.01
Fibrinogen, mmol/L	0.030 (0.028–0.031)	0.029 (0.027–0.030)	0.030 (0.029–0.032)	.001
Homocysteine, µmol/L^g^	7.8 (7.3–8.2)	8.6 (8.1–9.1)	6.9 (6.5–7.4)	< .001

Abbreviations: BMI, body mass index; 25(OH)D, 25-hydroxyvitamin D; HOMA-IR, homeostatic model assessment of insulin resistance; HDL-C, high-density lipoprotein cholesterol; LDL-C, low-density lipoprotein cholesterol; ApoA1, apolipoprotein A1; ApoB, apolipoprotein B; CRP, C-reactive protein.

a Values expressed as mean (95% confidence interval) unless otherwise indicated.

b All *P* values are for tests for differences between sexes. All tests are *t* tests except for hormone use and race, which are χ^2^ tests.

c Race groups were white (n = 1,636), Asian (n = 155), and other (n = 89). Information on race was missing for 48 (2.5%) participants.

d χ^2^ results for race (white and Asian groups only) by sex. Interpret with caution because of high sampling variability associated with Asian group.

e Marginal coefficient of variation (CV) denoting high sampling variability associated with estimate. Use with caution. CV was 27% for all, 31% for men, and 28% for women.

f Variables not normally distributed were square-root transformed for analyses.

g Variables not normally distributed were log transformed for analyses.

Mean plasma 25(OH)D levels varied by month of clinic visit among men and women (Figure). Concentrations of 25(OH)D were highest from June through August, and lowest in January and February in both sexes. Concentrations of 25(OH)D fluctuated seasonally in both men and women, but the extent of fluctuation observed was greater among men than women; plasma 25(OH)D concentrations in men ranged from 53 nmol/L to 80 nmol/L. Mean 25(OH)D concentrations in women ranged only from 62 nmol/L to 75 nmol/L.

**Figure:**
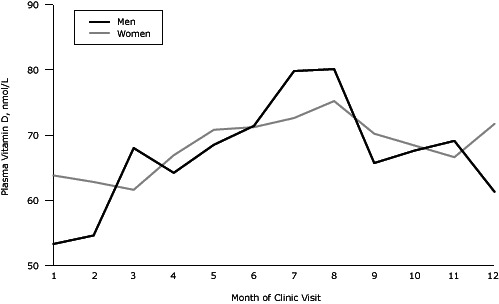
Mean plasma vitamin D, by month of clinic visit and sex, Canadian Health Measures Survey, 2007–2009. **Table:** 

Month of Clinic Visit	Plasma Vitamin D, nmol/L	
	Men	Women
1	53.3	63.8
2	54.6	62.8
3	68.0	61.6
4	64.2	66.9
5	68.5	70.8
6	71.4	71.2
7	79.8	72.6
8	80.1	75.2
9	65.7	70.2
10	67.6	68.4
11	69.1	66.6
12	61.3	71.7

Mean levels of cardiometabolic biomarkers by quartiles of plasma 25(OH)D are shown in Table 2. Significant inverse linear associations were noted between plasma 25(OH)D and insulin, HOMA-IR, triglycerides, total cholesterol, LDL-C, and total cholesterol:HDL-C. No significant linear associations with plasma 25(OH)D were noted for glucose, HDL-C, ApoA1, ApoB, CRP, fibrinogen, and homocysteine.

**Table 2:** Cardiometabolic Biomarkers, by Quartiles of Plasma 25-Hydroxyvitamin D (25[OH]D), Canadian Health Measures Survey, 2007–2009^a^

Biomarker	25(OH)D Quartiles				β^b^ (SE)	*P* Value^b^
	Q1 (n = 476)	Q2 (n = 477)	Q3 (n = 477)	Q4 (n = 476)		
**25(OH)D, nmol/L**						
Range	11.62–49.91	49.92–65.79	65.80–83.53	83.54–267.07	NC	NC
Mean (95% CI)	38.1 (36.6–39.6)	57.9 (57.1–58.7)	74.4 (73.6–75.1)	102.5 (98.6–106.5)	NC	NC
Glucose, mmol/L^c^	5.2 (5.0–5.3)	5.1 (5.0–5.3)	5.0 (4.9–5.2)	5.0 (4.8–5.1)	−0.02 (0.02)	.23
Insulin, pmol/L^c^	83.0 (67.8–98.3)	66.9 (59.8–74.0)	65.6 (57.4–73.9)	64.8 (57.7–72.0)	−0.16 (0.04)	.003
HOMA-IR^c^	2.9 (2.4–3.4)	2.3 (2.0–2.5)	2.2 (1.8–2.5)	2.1 (1.9–2.4)	−0.18 (0.05)	.002
Triglycerides, mmol/L^c^	1.5 (1.3–1.7)	1.3 (1.2–1.5)	1.3 (1.1–1.4)	1.2 (1.1–1.3)	−0.21 (0.06)	.004
Total cholesterol, mmol/L	4.9 (4.7–5.1)	5.0 (4.9–5.2)	4.8 (4.6–5.1)	4.6 (4.4–4.8)	−0.31 (0.14)	.05
HDL-C, mmol/L^d^	1.3 (1.2–1.3)	1.3 (1.3–1.4)	1.4 (1.3–1.5)	1.3 (1.3–1.4)	0.02 (0.01)	.13
LDL-C, mmol/L^d^	3.1 (2.9–3.3)	3.2 (3.0–3.3)	3.0 (2.8–3.1)	2.7 (2.6–2.9)	−0.09 (0.03)	.02
Total:HDL-C^c^	4.0 (3.7–4.3)	4.0 (3.8–4.3)	3.7 (3.5–3.9)	3.6 (3.4–3.8)	−0.09 (0.02)	.004
ApoA1, g/L^d^	1.4 (1.4–1.4)	1.4 (1.4–1.5)	1.5 (1.4–1.5)	1.4 (1.4–1.5)	0.02 (0.01)	.10
ApoB, g/L^c^	0.92 (0.86–1.00)	0.95 (0.91–0.98)	0.89 (0.85–0.94)	0.85 (0.82–0.88)	−0.07 (0.04)	.07
CRP, mg/L^c^	2.3 (1.7–2.9)	2.3 (1.9–2.6)	2.4 (2.1–2.7)	2.1 (1.7–2.4)	0.09 (0.13)	.52
Fibrinogen, mmol/L	0.030 (0.028–0.032)	0.030 (0.028–0.031)	0.030 (0.028–0.031)	0.029 (0.028–0.031)	0.01 (0.01)	.56
Homocysteine, µmol/L^c^	8.0 (7.2–8.8)	7.4 (7.0–7.9)	8.0 (7.4–8.6)	7.6 (7.0–8.2)	−0.04 (0.04)	.39

Abbreviations: SE, standard error; NC, not calculated; CI, confidence interval; HOMA-IR, homeostatic model assessment of insulin resistance; HDL-C, high-density lipoprotein cholesterol; LDL-C, low-density lipoprotein cholesterol; ApoA1, apolipoprotein A1; ApoB, apolipoprotein B; CRP, C-reactive protein.

a Shown are biomarker crude means (95% CI) for each 25(OH)D quartile (weighted).

b Regression coefficients (β), SEs, and *P* values for the linear relationship between 25(OH)D and biomarkers adjusted for sex, waist circumference, physical activity, season, and hormone drug use.

c Where necessary, variables were natural log-transformed for analysis.

d Where necessary, variables were square-root transformed for analysis.

The relationship between 25(OH)D and cardiometabolic biomarkers in men and women separately is shown in Table 3. Among men, plasma 25(OH)D was inversely associated with insulin, HOMA-IR, and triglycerides. Among women, plasma 25(OH)D was inversely associated with insulin, HOMA-IR, total cholesterol, LDL-C, and total cholesterol:HDL-C. We noted a trend suggesting that 25(OH)D may be a stronger predictor of fasting insulin levels and HOMA-IR among men than women (insulin: β= −0.21 among men vs −0.13 among women; HOMA-IR: β= −0.24 among men vs −0.14 among women). However, for both of these biomarkers, the 95% confidence intervals for the β coefficients from each sex overlapped (data not shown), indicating that the trend did not reach significance.

**Table 3:** Linear Regression Examining Cardiometabolic Biomarkers and Plasma 25-Hydroxyvitamin D (25[OH]D), by Sex, Canadian Health Measures Survey, 2007–2009^a^

Biomarker	Men		Women	
	β^b^ (SE)	*P* Value^b^	β^b^ (SE)	*P* Value^b^
Glucose, mmol/L^c^	−0.03 (0.02)	.32	−0.02 (0.02)	.35
Insulin, pmol/L^c^	−0.21 (0.05)	.002	−0.13 (0.06)	.05
HOMA-IR^c^	−0.24 (0.06)	.002	−0.14 (0.06)	.03
Triglycerides, mmol/L^c^	−0.26 (0.09)	.01	−0.15 (0.08)	.09
Total cholesterol, mmol/L	−0.27 (0.16)	.12	−0.34 (0.15)	.05
HDL-C, mmol/L^d^	0.009 (0.01)	.45	0.02 (0.02)	.27
LDL-C, mmol/L^d^	−0.08 (0.04)	.09	−0.10 (0.04)	.02
Total:HDL-C^c^	−0.07 (0.05)	.13	−0.10 (0.02)	.001
ApoA1, g/L^d^	0.008 (0.01)	.42	0.02 (0.01)	.17
ApoB, g/L^c^	−0.07 (0.04)	.15	−0.07 (0.04)	.10
CRP, mg/L^c^	0.04 (0.18)	.82	0.10 (0.14)	.52
Fibrinogen, mmol/L	−0.01 (0.01)	.28	0.01 (0.01)	.19
Homocysteine, µmol/L^c^	−0.04 (0.04)	.33	−0.03 (0.06)	.61

Abbreviations: SE, standard error; HOMA-IR, homeostatic model assessment of insulin resistance; HDL-C, high-density lipoprotein cholesterol; LDL-C, low-density lipoprotein cholesterol; ApoA1, apolipoprotein A1; ApoB, apolipoprotein B; CRP, C-reactive protein.

a Shown are values for the linear relationship between 25(OH)D and biomarkers for men and women separately.

b Linear regression adjusted for waist circumference, physical activity, season, and hormone drug use.

c Where necessary, variables were natural log-transformed for analysis.

d Where necessary, variables were square-root transformed for analysis.

## Discussion

We examined the association between vitamin D and traditional and novel biomarkers of cardiometabolic disease and found that it was inversely associated with insulin, insulin resistance, triglycerides, total cholesterol, LDL-C, and the ratio of total cholesterol to HDL-C, but not with fasting glucose, ApoA1, ApoB, CRP, fibrinogen, or homocysteine. This pattern varied between men and women.

We recently reported an inverse association between plasma 25(OH)D and metabolic syndrome risk (12) and obesity (13) in Canadians. However, to our knowledge, this study is the first to examine the association between vitamin D and individual biomarkers of cardiometabolic risk in the general Canadian population. We observed inverse associations between 25(OH)D and several traditional cardiometabolic risk factors, such as insulin, HOMA-IR, and biomarkers of lipid metabolism. Our findings are in agreement with previous reports that have associated low 25(OH)D with worsened levels of biomarkers of glycemic control and lipid metabolism (12,16). Considering the widespread prevalence of vitamin D insufficiency in Canada and other countries around the world, understanding the degree to which low nutritional status of this vitamin affects disease-associated processes is key toward informing recommendations, both at the individual and the population levels, to obtain adequate vitamin D. Although our findings are cross-sectional, together with previous research conducted in Canada and elsewhere (8,12,16–18) they suggest that an adequate vitamin D status may help prevent the development of cardiometabolic disease-related processes.

Evidence from in vitro studies suggests a role for vitamin D in improving insulin secretion and sensitivity (19,20). However, associations between 25(OH)D and markers of glycemic control in humans have been inconsistent (16,17,21,22). The discrepancies may result from small sample sizes and from differences in racial background and disease status of the subjects assessed. We recently reported an inverse association between 25(OH)D and insulin resistance, as measured by HOMA-IR, in people without diabetes from the general Canadian population (12). As this study indicates, the inverse association between 25(OH)D and HOMA-IR is also present when people with diabetes are included in the analysis. Overall, these observations suggest a role for 25(OH)D in modulating glycemic responses, both in healthy people and in those who have impaired glycemic control. These findings may have public health implications and suggest that vitamin D supplementation may be used both in cardiometabolic disease prevention and in improving its complications. We noted a nonsignificant trend suggesting that 25(OH)D may be a stronger predictor of glycemic control biomarkers among men than women. This sex-based difference may be more apparent in populations with a greater incidence of cardiometabolic disease.

In this study, we reported inverse associations between 25(OH)D and triglycerides, total cholesterol, LDL-C, and total cholesterol:HDL-C in the overall population. When examined separately by sex, 25(OH)D was associated with triglycerides only among men. However, we also observed an inverse trend between 25(OH)D and triglycerides among women, although it failed to reach significance. Conversely, total cholesterol, LDL-C, and total cholesterol:HDL-C were inversely associated with 25(OH)D only among women. Again, similar inverse trends were observed among men, although they did not reach significance. Similar to our findings, the findings of many studies have reported inverse associations between 25(OH)D and various serum lipids (18,21,23). Although the mechanistic link between 25(OH)D and lipid metabolism remains poorly understood, vitamin D has been proposed to modulate the transcription activity of an array of genes known to be involved in lipid metabolism (24,25). In addition, previous research suggests that vitamin D may upregulate lipoprotein lipase activity in adipocytes, which would, in turn, result in decreased circulating triglyceride levels (26).

The consensus of available information from animal and cell culture models suggests that vitamin D modulates immune responses and may ameliorate inflammatory states (27). However, the association between serum 25(OH)D and biomarkers of inflammation, such as CRP and fibrinogen, in epidemiologic and clinical studies in humans has been inconsistent (28,29). In this population-based cohort, after adjusting for relevant covariates, we observed no association between 25(OH)D and inflammatory biomarkers, either in the population as a whole or for either sex. However, it is possible that the anti-inflammatory effects of vitamin D are observed only in severely vitamin D-deficient individuals or those with an immunocompromised status (3).

Previous studies have observed differences in levels of 25(OH)D by sex as well as differences in seasonal variation of circulating 25(OH)D (9,11). Our results confirm these sex-based differences in seasonal fluctuations; men showed a greater degree of fluctuation than women (53–80 nmol/L vs 62–75 nmol/L, respectively). Although these differences can be a result of biologic or lifestyle factors, they may implicate a sex-related disparity in response to vitamin D intake. Elucidating whether these sex-associated month-to-month differences in vitamin D levels translate into different effects of vitamin D on disease-related processes in men and women warrants further study.

Our study has limitations. Although representative of the racial distribution of the Canadian population, the small sample size in the nonwhite group prevented examining the associations assessed in this study in other race groups living in Canada. Other studies have reported variation in 25(OH)D levels across races, despite a similar intake of vitamin D intake from diet and supplements (9,30). Moreover, we have shown that Asian Canadians have significantly lower serum 25(OH)D than their white counterparts (12). Understanding the relationship between 25(OH)D and cardiometabolic risk factors across race groups in Canada may help develop intervention strategies targeted to vulnerable subgroups (ie, at-risk populations). Furthermore, we were unable to consider the potential effects on the associations we investigated of parathyroid hormone, calcium, phosphate, and fibroblast growth factor 23, all of which play central roles in vitamin D metabolism (10). Although waist circumference and physical activity are considered good indicators of visceral adiposity and fitness, respectively, we lacked direct measurements of the latter variables, which may play roles in the link between vitamin D and cardiometabolic disease. Finally, residual confounding and unaccounted influences of diet or genetic variations may affect some of the associations reported here.

We found inverse associations between 25(OH)D and biomarkers of cardiometabolic disease risk in a population-based cohort representative of Canadian adults. We also observed month-to-month fluctuations in 25(OH)D and noted that plasma 25(OH)D levels fluctuated more widely in men than women. Our results contribute to an increasing body of evidence suggesting that vitamin D modulates processes associated with risk of cardiometabolic disease at the population level. These findings may have public health implications for recommending vitamin D to prevent cardiometabolic disease and related conditions.
